# The Role of Policy in Preventing Discrimination-Based Suicide and Substance Use Coping Outcomes Within the Transgender Community

**DOI:** 10.1007/s11121-025-01825-8

**Published:** 2025-07-08

**Authors:** Patrick O’Neill, Whitney Becker, Casey A. Cunningham, Damon E. Jones, Ashley N. Linden-Carmichael

**Affiliations:** 1 Department of Human Development and Family Studies, The Pennsylvania State University, University Park, PA USA; 2 Evidence-to-Impact Collaborative, The Pennsylvania State University, University Park, PA USA; 3 Department of Psychology, North Carolina State University, Raleigh, NC USA; 4 Edna Bennett Pierce Prevention Research Center, The Pennsylvania State University, University Park, PA USA; 5 Department of Counseling and Human Services, University of Oregon, Eugene, OR USA

**Keywords:** Transgender/gender diverse communities, Health care discrimination, Suicide, Substance use coping, Public policy

## Abstract

Individuals who identify as transgender/gender diverse face an increased risk of discrimination, particularly in health care settings. This discrimination has been associated with higher odds of substance use coping and attempting suicide. However, risk and protective factors that can moderate this relationship remain under-evaluated. The present study examines the role of LGBTQ-friendly state policy climates in preventing discrimination-based outcomes of substance use coping and attempting suicide in the transgender/gender diverse community. Data from the 2008–2009 National Transgender Discrimination Survey were merged with state policy climate scores. Policy climate scores were derived from the tracking of legislation through the Movement Advancement Project. Participants were asked about using substances to cope, prior suicide attempts, and three types of discrimination at mental health clinics (denied equal treatment, verbal harassment/disrespect, physical assault). Being denied equal treatment or service and being verbally harassed or disrespected were both associated with increased odds of using substances to cope and attempting suicide. Residing in a more LGBTQ-friendly policy climate moderated the relationship between being denied equal treatment and a prior suicide attempt as well as the relationship between verbal harassment and both suicide attempt and use of substances to cope. Findings underscore the potential of LGBTQ-friendly policy climates to serve as a protective factor against discrimination faced by the transgender/gender diverse community. A greater understanding of the role of policies in the relationship between discrimination and adverse outcomes can help form policy-based prevention in an effort to improve the lives of individuals in the transgender/gender diverse community.

**Supplementary Information:**

The online version contains supplementary material available at 10.1007/s11121-025-01825-8.

## Introduction

Individuals who identify as transgender/gender diverse (TGD), including individuals who identify as binary and non-binary as well as transgender and gender diverse individuals, are at increased risk for experiencing discrimination including microaggressions, sexual harassment, and physical violence (Casey et al., 2019). Discrimination can occur in numerous contexts including within the family unit (de Lange et al., 2022), in public accommodations (Lee et al., 2016), and in health care settings (Johns et al., 2023; Kattari et al., 2015). Discrimination in health care settings is of particular concern because it can prevent people who identify as TGD, especially individuals in the Black, Indigenous, and People of Color (BIPOC) communities, from utilizing protective health services including mental health care clinics (Kattari et al., 2015; Wylie et al., 2016). There is evidence to suggest individuals in the TGD community experience higher levels of mental health concerns relative to cisgender individuals (Pellicane & Ciesla, 2022; Su et al., 2016; Vargas et al., 2020), and their experiences of discrimination in health care settings can have negative subsequent effects on their wellbeing (Johns et al., 2023; Kattari et al., 2015). Therefore, understanding the impact of these discriminatory experiences, and potential preventative measures to mitigate their impact, is of critical importance.

Discrimination is a significant risk factor for mental health concerns, including depression, among individuals in the TGD community (Khobzi Rotondi, 2012). Both depression and gender-based discrimination are longitudinally associated with suicide attempts (Clements-Nolle et al., 2006) and other maladaptive outcomes, including substance use. Individuals who identify as TGD are at heightened risk for reporting using substances to cope with discrimination experiences (Clark, 2014; Felner et al., 2020); specifically, individuals from the TGD community are nearly 50% more likely than cisgender individuals to engage in substance use throughout the lifespan (Cotaina et al., 2022). Additionally, gender-based discrimination is linked to cannabis use disorder (Lee et al., 2016), nicotine use disorder (Slater et al., 2017), and heavy alcohol use (Slater et al., 2017).

The links between discrimination, substance use, and mental health concerns, including suicide attempts, can be better understood through the lens of the Minority Stress Model (Meyer, 1995, 2003). This model posits that chronic factors exist in various social environments (e.g., in public, at the doctor’s office) that are unique to minoritized individuals, including gender and sexual minorities. These systemic factors include experiences and beliefs such as stressful events, expectations of rejection, and internalized stigma, which can exacerbate stress levels for minoritized individuals. In particular, health care settings can become stressful environments if people who identify as TGD face discrimination in those locations (Bockting et al., 2013).

While stressful environments and events, such as health care discrimination, can serve as negative factors that augment stressors for TGD communities, there are a myriad of positive factors that can help mitigate stressors. Inclusive social environments where people who identify as LGBTQ + can feel safe and affirmed in who they are (e.g., environments that value diverse viewpoints and places with gender neutral bathrooms) can provide a safeguard against minority stress resulting from the negative experiences and events (Gower et al., 2019; Painter et al., 2018). Additionally, a recent survey from The Trevor Project found that high familial social support, accepting and affirming school environments, and living in LGBTQ-accepting communities are linked to lower rates of attempting suicide (The Trevor Project, 2022). These environments are created, at least in part, by LGBTQ-friendly public policies that can have a direct effect on the wellbeing of people who identify as TGD. Prior research suggests that state-level anti-transgender policies (e.g., North Carolina’s “Bathroom bill” (North Carolina General Assembly, 2016)) and the banning of gender-affirming medical care for certain communities) may have adverse effects on youth in the TGD community, with 93% of youth in the TGD community reporting concerns regarding being denied access to affirming medical care due to state and/or local laws (The Trevor Project, 2022). However, public policy can also have a positive impact on people who identify as TGD. States and localities with anti-discrimination policies are associated with people in the TGD community accessing gender-affirming health care at higher rates (Du Bois et al., 2018). Additionally, states implementing policies banning gender-based discrimination reported lower odds of suicidality for gender minorities in the years following policy implementation (McDowell et al., 2020).

## Present Study

Individuals in the TGD community experience increased levels of discrimination compared to cisgender individuals (Casey et al., 2019). Considering the increased rates of mental health concerns they face, specifically substance use coping and suicidality (Clark, 2014; Cotaina et al., 2022; Pellicane & Ciesla, 2022; Su et al., 2016; Vargas et al., 2020), examining discrimination in health care settings, including mental health clinics, in the TGD community is of paramount importance (Clements-Nolle et al., 2006; Vargas et al., 2020).

Additionally, policy climates may be indicative of structural stigma, and it is well-documented that individuals who live in places with lower structural stigma experience improved health outcomes, including a lower risk of suicidality (Perez-Brumer et al., 2015). Individuals who identify as LGBTQ who live in a state with non-negative policy climates may experience a stronger sense of community belonging (Chai, 2024), which may serve as a buffer to experiences of discrimination and subsequent coping behavior. Indeed, state-level policies and associated stigma have been documented to have both main and moderating effects on experiences of discrimination (Clark et al., 2022) as well as links between relevant experiences during adolescence (e.g., gender affirming medical care) on adult psychological distress (Lee et al., 2024). Consequently, the goal of the current study was to examine whether the impact of discrimination had a differential impact on health outcomes based on state-level policies. Our study had two research questions:

1. Do LGBTQ-friendly state-level policies moderate the relationship between discriminatory experiences at mental health clinics in TGD communities and suicide attempts?
2. Do LGBTQ-friendly state-level policies moderate the relationship between discriminatory experiences at mental health clinics in TGD communities and substance use-related coping?

We hypothesized that LGBTQ-friendly policy climates would attenuate the effect of discriminatory mental health clinic experiences on the odds of substance use coping and suicide attempts among individuals in the TGD community.

## Method

Data were from the 2008–2009 version of the National Transgender Discrimination Survey, a questionnaire administered by the National Gay and Lesbian Task Force and the National Center for Transgender Equality (Grant et al., 2020). During the creation of this survey, questions were created in consultation with members of the transgender community who used their lived experiences to help guide the framing of each question. For the present study, as we were only able to track state-level policy climates in the USA, our sample (*N* = 6141) was restricted to only individuals who resided in the USA. A population density display of the sample can be found in the supplementary material (see Supplementary Fig. 1).

Data collection procedures for the National Discrimination Survey were approved by the university’s institutional review board. Participants provided informed consent and were asked to complete a battery of questionnaires. In addition to survey data, state-level LGBTQ-friendly policy climate scores from 2010 were derived from the Movement Advancement Project (MAP; Movement Advancement Project, 2020).^1^

### Measures

#### Mental Health Clinic Discriminatory Experiences

Participants were asked, “Based on being transgender/gender non-conforming, please check whether you have experienced any of the following in these public spaces” with a variety of environments listed. Within each environment, they had the option to individually check boxes for “Denied equal treatment or services,” “Verbally harassed or disrespected,” and “Physically attacked or assaulted” if they had experienced these types of discrimination at any point in their life. The current study focused on experiences in mental health clinics; questions concerning other environments (e.g., Doctor’s office or hospital) were omitted.

### Suicide Attempt

Participants were asked if they had ever previously attempted suicide, coded as [1] Yes or [2] No.

### Substance Use Coping

Participants were provided with the prompt, “I drink or misuse drugs to cope with the mistreatment I face or faced as a transgender or gender non-conforming person” and could self-report either [1] Yes, [2] Yes, but not currently, [3] No, or [4] Not applicable, I face no mistreatment. Responses 1 and 2 were collapsed to indicate (1) substance.

use coping at any time as a result of discrimination, and levels three and four were combined to indicate (0) no substance use coping.

### Racial-Ethnic Identity

Participants were able to check all racial-ethnic groups with which they identified. A full breakdown of how participants identified can be found in Table 1. Preliminary analyses revealed the sample was underpowered to identify individual differences across racial-ethnic groups, and thus, race-ethnicity was dichotomized such that participants were coded as identifying as White (if any of their self-identified racial-ethnic groups was white) or BIPOC.^2^

**Table 1:** Demographic description of participants

Variable	*M* (*SD*) or *n* (%)
**Age**	36.85 (13.14)
**Race**	
White	5146 (83.8%)
Black or African American	358 (5.8%)
American Indian or Alaska Native	355 (5.8%)
Hispanic or Latino	377 (6.1%)
Asian or Pacific Islander	200 (3.3%)
Arab or Middle Eastern	45 (0.7%)
Multiracial	464 (7.6%)
**Had health insurance**	4980 (81%)
**Education level**	
Less than high school	231 (4%)
High school diploma/GED	507 (8%)
Some college	1702 (28%)
Associates or technical degree	771 (13%)
College degree	1676 (27%)
Graduate/professional degree	1223 (20%)
Previously attempted suicide	2495 (41%)
Reported using substances to cope	1585 (26%)
**State policy climate scores**	
Negative climate	27 (54%)
Non-negative climate	23 (46%)

*Note*. Education level: missing *n* = 31

### Education

Participants were asked for their highest level of education completed with options including [1] less than high school, [2] high school/GED, [3] some college, [4] associates or technical school degree, [5] college degree, and [6] graduate or professional degree.

### Health Insurance

Participants were asked about health insurance status (i.e., private, public, or no insurance). Responses were collapsed into one variable that indicated whether a participant had any type of health insurance (i.e., 0 = participant did not have health insurance, 1 = participant had health insurance).

### Age

Participants were voluntarily asked to record their age in years in the demographic part of the questionnaire.

### LGBTQ-Friendly State Policy Climate

LGBTQ-friendly state-level policy climate scores were derived from MAP (Movement Advancement Project, 2020). MAP tracks over 50 LGBTQ-related laws and policies at the state level and assigns points to each state for laws that are enacted, resulting in a single summed score representing the policy climate of an individual state. Additionally, states received negative points for each anti-LGBTQ law they enacted that was tracked. In total, across the policies tracked by MAP, policy climate scores ranged from − 13 (Tennessee) to 43 (Colorado). States were assigned a categorical value (negative [1], low [2], fair [3], medium [4], high [5]) depending on the percentage of total available points they received (< 0 points, 0–24.9% of points, 25–49.9% of points, 50–74.9% of points, and 75–100% of points, respectively), which indicated the degree of LGBTQ-friendly policies for each state. In the 2010 report (Movement Advancement Project, 2020), states received varying categorical ratings: 27 had negative values, 10 had low values, 10 had fair values, and three had medium values.^1^ As the current study aimed to examine the potential detrimental implications of living in the most negative policy climate, we combined low, fair, and medium values into a “non-negative” category. As a result, 27 states were coded as negative policy climates, and 23 states were coded as non-negative policy climates.

### Statistical Analysis

Given the nested nature of the data at the state level and focus on state-level policies, multilevel modeling was evaluated as a means of analysis, but preliminary analysis revealed the intraclass correlations (ICCs) were 0.0058 and 0.0095 for the substance use coping and suicide attempt outcomes, respectively. In other words, less than 1% of variability in each outcome was accounted for by the states, leaving over 99% to be accounted for by individual factors. Thus, we conducted main effect logistic regression models to examine the association between experiences at mental health clinics and (1) odds of a lifetime suicide attempt and (2), separately, odds of using substances to cope. LGBTQ-friendly policies were added as a moderator of each association and were followed up by conducting simple slope analyses (plots found in Supplementary Fig. 2). In all analyses, we controlled for racial-ethnic group, education level, population level, participant age, and health insurance status. White participants with no health insurance and a less than high school education who lived in states with a “negative” policy climate score served as the reference group for analyses. All analyses were conducted within R Studio (RStudio Team, 2022).

## Results

Descriptive statistics are shown in Table 1. Notably, almost half of the sample population (*n* = 2495) reported having attempted suicide while roughly one-quarter (*n* = 1585) reported using substances to cope with the discrimination they faced.

Results of the main effect models for both outcome variables are presented in Table 2. Compared to individuals who did not face discrimination at mental health clinics, individuals who identified as TGD and reported being denied equal treatment had two times the odds of engaging in substance use-related coping (*p* < 0.001) and nearly three times the odds of attempting suicide (*p* < 0.001). Additionally, being verbally harassed or disrespected at a mental health clinic increased the odds of using substances to cope by a factor of 1.31 (*p* = 0.03) and the odds of a suicide attempt by a factor of 2.69 (*p* < 0.001). Being physically attacked or assaulted was not significantly associated with odds of coping via substance use (*p* = 0.68) or attempting suicide (*p* = 0.72). Living in a state with a non-negative policy climate did not increase the odds of coping via substance use (*p* = 0.20) or attempting suicide (*p* = 0.70) for TGD-identifying individuals. Individuals who identified as both BIPOC and TGD were also at higher odds of attempting suicide and using substances to cope. Having health insurance and having a college or graduate degree were associated with lower odds of both outcomes. Age was not a significant predictor of substance use coping. However, TGD-identifying individuals were significantly less likely to attempt suicide for each additional age (*OR* = 0.992).

**Table 2:** Regression model of the effects of discriminatory mental health clinic experiences

Predictors	**Odds Ratio (95% CI) **	
	**Using substances to cope**	**Suicide attempt**
Denied equal treatment or service	1.97 (1.54, 2.51)***	2.88 (2.24, 3.73)***
Verbally harassed or disrespected	1.31 (1.03, 1.67)*	2.69 (2.10, 3.48)***
Physically attacked or assaulted	1.18 (0.55, 2.57)	0.86 (0.39, 1.99)
Non-negative policy climate score	1.09 (0.95, 1.25)	0.98 (0.87, 1.10)
BIPOC	1.28 (1.10, 1.49)**	1.33 (1.16, 1.52)***
Having health insurance	0.73 (0.63, 0.86)***	0.74 (0.64, 0.85)***
Highest education level	0.88 (0.84, 0.92)***	0.83 (0.80, 0.87)***
Age	1.00 (0.99, 1.003)	0.992 (0.987, 0.997)***

*Note*. ^*^p <.05, **p <.01, ***p <.001; reference/intercept group: transgender individuals who are White/non-minoritized group, do not have health insurance, completed less than high school, and live in negative policy climate states

The results of the moderation models can be seen in Table 3. Experiences of being denied equal treatment and verbal harassment or disrespect were associated with significantly higher odds of using substances to cope, with odds of 2.25 and 1.75, respectively. Residing in a state with a non-negative policy climate was associated with increased odds of coping via substance use (*p* = 0.03). Compared to TGD-identifying individuals in states with negative policy climate scores (OR = 1.11, 95% CI = [0.76, 1.62]), TGD-identifying individuals in states with non-negative policy scores had weaker associations (OR = 0.93, 95% CI [0.75, 1.15]) between being physically attacked or assaulted and using substances to cope. Additionally, TGD-identifying individuals who were denied equal treatment or verbally harassed or disrespected had significantly higher odds of attempting suicide (5.14 and 5.88 respectively). Relative to TGD-identifying individuals who resided in states with negative policy climate scores (OR = 1.43, 95% CI = [1.28, 1.59]), TGD-identifying individuals who lived in non-negative policy climates (OR = 1.22, 95% CI [1.14, 1.30]) had weaker associations between experiences of being denied equal treatment or service and suicide attempts. Finally, TGD-identifying individuals who resided in states with non-negative (OR = 1.16, 95% CI = [1.08, 1.24])—relative to negative policy climates (OR = 1.47, 95% CI = [1.33, 1.63])—had an attenuated relationship between the experience of being verbally harassed or disrespected and suicide attempts (see Supplementary Fig. 2).

**Table 3:** Regression model of the effects of discriminatory mental health clinic experiences with covariates and moderators

Predictors	**Odds Ratio (95% CI) **	
	**Using substances to cope**	**Suicide attempt**
Denied equal treatment or service	2.25 (1.49, 3.43)***	5.14 (3.11, 8.91)***
Verbally harassed or disrespected	1.75 (1.16, 2.63)**	5.88 (3.65, 9.91)***
Physically attacked or assaulted	7.69 (1.34, 145.44)	1.59 (0.31, 11.93)
Non-negative policy climate score	1.17 (1.01, 1.35)*	1.07 (0.95, 1.21)
BIPOC	1.28 (1.10, 1.49)**	1.32 (1.15, 1.51)***
Have health insurance	0.74 (0.63, 0.87)***	0.74 (0.64, 0.85)***
Highest education level	0.88 (0.84, 0.92)***	0.83 (0.80, 0.86)***
Age	0.998 (0.993, 1.004)	0.991 (0.987, 0.996)***
Denied equal treatment or service*policy climate	0.83 (0.50, 1.39)	0.46 (0.25, 0.83)*
Verbally harassed or disrespected*policy climate	0.63 (0.38, 1.05)	0.33 (0.18, 0.57)***
Physically attacked or assaulted*policy climate	0.09 (0.004, 0.64)*	0.47 (0.05, 3.06)

*Note*. ^*^p <.05, **p <.01, ***p <.001; reference/intercept group: transgender individuals who are White/non-minoritized group, do not have health insurance, completed less than high school, and live in negative policy climate states

## Discussion

The present study aimed to increase understanding of the role of LGBTQ-friendly political climates in the relationship between discriminatory mental health clinic experiences and substance use coping and suicide attempt outcomes in a nationally representative sample of individuals who identify as TGD. This study found that being denied equal treatment or service and being verbally harassed or disrespected were both associated with increased odds of substance use coping and attempting suicide while being physically attacked or assaulted was not associated with either outcome. Additionally, individuals who identified as BIPOC experienced higher odds of both outcomes (i.e., attempting suicide and substance use coping); additionally, individuals with a higher level of education and with health insurance experienced lower odds of both outcomes. Overall, the results of this study illustrate the barriers individuals who identify as TGD may face when seeking mental health treatment and the compounding adverse effects these experiences may have on their health and well-being, including using substances to cope or attempting suicide.

Compared to individuals who lived in states with a negative policy climate score, individuals who lived in states with more favorable (non-negative) policies generally had weaker associations between being denied equal treatment or service, experiencing verbal harassment, being physically attacked or assaulted, and using substances to cope or a suicide attempt. Specifically, individuals who resided in states with non-negative policy scores relative to negative scores had weaker associations between both being verbally harassed/disrespected and being denied equal treatment/service and attempting suicide. Individuals living in states with non-negative policy climate scores also had weaker associations between being physically attacked or assaulted and using substances to cope. Overall, these results suggest that individuals who live in more LGBTQ-friendly policy climates experience weaker associations between discriminatory experiences at mental health clinics and odds of suicide attempts and using substances to cope.

These findings are consistent with existing literature regarding the overall influence of LGBTQ-related policies on the relationship between discrimination and adverse outcomes for gender minority communities (Du Bois et al., 2018; Rabasco & Andover, 2020; Russell et al., 2010; Watson et al., 2021). However, current research has also shown conflicting results over the impact of specific types of policies (i.e., inclusive and protective policies; McDowell et al., 2020). Previously, inclusive LGBTQ policies were associated with greater substance use (in the form of binge drinking) while the impact of protective policies on reducing health inequities was mixed (Watson et al., 2021). As the authors suggest, Watson et al.’s (2021) findings surrounding inclusive LGBTQ policies and increased substance use, the first of their kind to be documented, may be due to increased socialization and social trust among youth peers within their own communities and not as a direct result of the policies. Meanwhile, Rabasco and Andover (2020) showed protective legislation can moderate the relationship between stressors (i.e., victimization and discrimination) and suicide attempts in a transgender and gender-diverse sample. It is also worth noting that the implementation of LGBTQ policies or laws does not automatically equate to the prevention of adverse outcomes for gender minority communities. In the 12-month period following the enactment of a protective policy in Massachusetts (Massachusetts General Laws c.151B, n.d.), 65% of gender minority adults surveyed in the state reported experiencing discrimination that was subsequently associated with increased risks of negative emotional and physical symptoms (Reisner et al., 2015).

Findings from the current study suggest that additional steps may need to be taken to integrate these LGBTQ-friendly policies into organizations, such as mental health clinics, in order to prevent adverse outcomes. For example, education and training for mental health clinic staff may be helpful to ensure equitable treatment for TGD communities. Additionally, a review of the organizational anti-discrimination policy may also be warranted to eliminate verbal harassment experiences at mental health clinics (Eisenberg et al., 2020). Previous intervention research aimed at medical education programs indicates various types of interventions yield positive effects, including greater knowledge of LGBTQ health care concerns and increased levels of comfort in treating patients who identify as LGBTQ (Morris et al., 2019). Considering public policy through the lens of the Minority Stress Model (Meyer, 1995, 2003), the present study’s findings, among others (e.g., McDowell et al., 2020; Rabasco & Andover, 2020; Russell et al., 2010; Watson et al., 2021), highlight public policy as a mechanism of the Minority Stress Model that can provide support for those who identify as TGD. One should approach this finding with caution, however, as Testa et al. (2015) point out in the Gender Minority Stress Model (Testa et al., 2015), where they argue cis-normative rules and expectations that are imbedded throughout society can manifest in structural-level policies. Therefore, legislation has the potential to act as a stressor for TGD communities when it is discriminatory in nature and targeted at gender minorities (Crasnow, 2021; Schanzle et al., 2023; The Trevor Project, 2022). Therefore, when considering public policy as it relates to TGD communities, it is important to understand that its impact may fluctuate. While discriminatory policy environments may cause undue harm, our findings suggest more positive, supportive policy environments may serve as a protective factor for TGD communities against adverse outcomes.

### Limitations and Future Directions

There are limitations that should be noted when interpreting study results. First, data from the National Transgender Discrimination Survey (Grant et al., 2020) may be somewhat outdated (data are from 2008 to 2009), and a more recent version of the dataset (2015) does not include questions concerning using substances to cope. Furthermore, the nature of the current study precludes us from examining the temporality of study associations, and thus, it is unclear whether experiences of discrimination preceded the use of substances to cope or suicide attempts or vice versa. Findings represent general co-occurrence rather than potential causal implications. Future longitudinal work would benefit from examining temporal associations between study variables.

Additionally, while accounting for policies geared towards both sexual orientation and gender identity, the MAP data included in the present study does not specify policy climates specific to TGD communities. Rapid societal shifts in social perceptions and attitudes (Bolt, 2014; Seale et al., 2020) as well as LGBTQ-related policy climates have occurred during the last 15 years. Despite a recent wave of anti-transgender legislation including the banning of gender-affirming care for minors and restriction of transgender females’ participation in women’s sports in some states (Horne et al., 2022; Turban et al., 2021), more states are now rated as having more LGBTQ-friendly climates than at the time of this survey’s administration (2008–2009). In 2020, six states had “medium” climates while 15 had “high” (i.e., more positive) climates compared to three and zero, respectively, in 2010 (Movement Advancement Project, 2020). During that period (i.e., 2008–2009), over half of states were rated as having negative policy climate scores while zero had high scores. In just over a decade since, only eight states now have negative policy scores while 15 have high scores (Movement Advancement Project, 2020). Despite the recent anti-transgender legislation, these policy climate scores suggest there may be a shift towards policies that are more inclusive and protective of people who identify as TGD and broader LGBTQ communities. Future research understanding the current impact of LGBTQ + friendly policies on experiences in mental health clinics and health outcomes is strongly warranted, as is further evaluation of gender-affirming strengths-based approaches to engagement with the legislative sphere on related policies.

Another limitation of the present study concerns our focus on three types of discrimination at mental health clinics. Individuals who identify as TGD may face gender-based discrimination in other health care settings as well. Future research would benefit from evaluating the impact of policy on the relationship between discriminatory experiences in multiple different health care settings and adverse outcomes (e.g., substance use coping, suicidality). Additionally, future research would do well to further investigate findings from the present study that are less clear (e.g., the lack of policy climates’ main effects on adverse outcomes such as attempting suicide, despite their moderating effects).

## Conclusion

The present study highlights the important role of LGBTQ-friendly state policy climates on the lives of individuals in the TGD community and their potential to reduce or prevent adverse outcomes by moderating the effect of discriminatory mental health clinic experiences on health and well-being outcomes. Further understanding the role of policymaking in the relationship between discriminatory health care experiences (i.e., discrimination, harassment, physical assault) and adverse outcomes (i.e., substance use coping, suicidality) can help to form policy-focused prevention efforts. These policy-focused prevention efforts can have wide-ranging impacts in their bid to improve the lives of individuals who identify as TGD.

## Supplementary Information

Below is the link to the electronic supplementary material.

ESM 1 (397 KB DOCX)

ESM 2 (61.0 KB DOCX)

## Data Availability

Data from the National Transgender Discrimination Survey used in this study has historically been available through the Interuniversity Consortium for Political and Social Research (ICPSR) at https://www.icpsr.umich.edu/web/ICPSR/studies/37888, however it looks like the data has been removed since it was used for this study. Policy climate data can be found here: https://www.lgbtmap.org/equality-maps.

## Footnotes

1 It should be noted that 2010 is the latest available data on state-level LGBTQ-friendly policy 1 climate scores.

2 While it was not ideal to group all non-White participants into one value, recoding the variable in this way follows previous research suggesting BIPOC status, broadly defined, is associated with lower rates of psychological well-being and higher rates of substance use coping (Tao et al., 2022).
