# Supplementary figures and images for: The Role of Policy in Preventing Discrimination-Based Suicide and Substance Use Coping Outcomes Within the Transgender Community
Source: Prev Sci. 2025 Jul 8;26(6):899–907. doi: 10.1007/s11121-025-01825-8 (PMC12394276; doi:10.1007/s11121-025-01825-8)

**Supplementary Figure 1.** Sample population density map


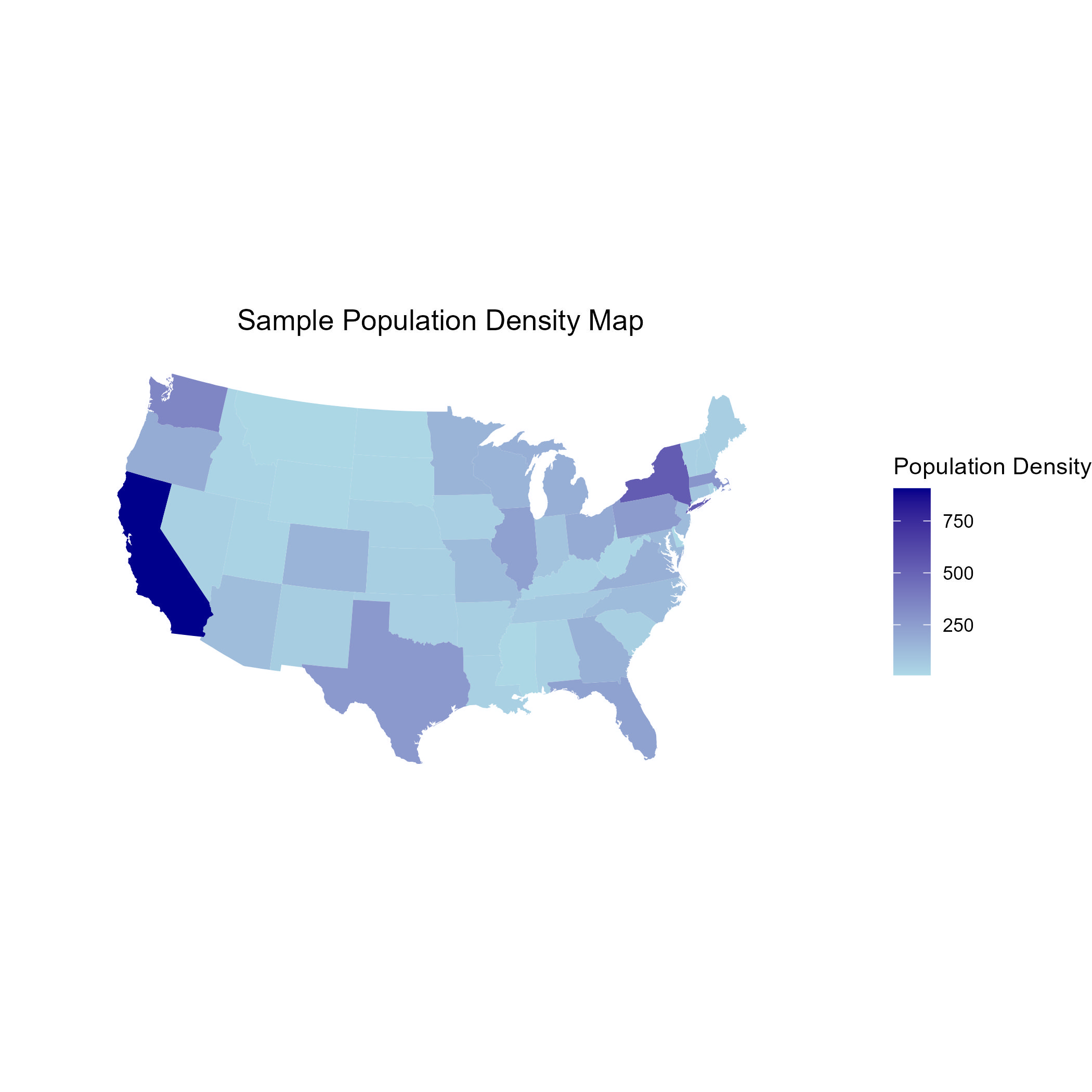

Supplement: Supplementary file 1 — (397 KB DOCX) [file 11121_2025_1825_MOESM1_ESM.docx]

**Supplementary Figure 2.** Simple slope plots of significant moderation effects


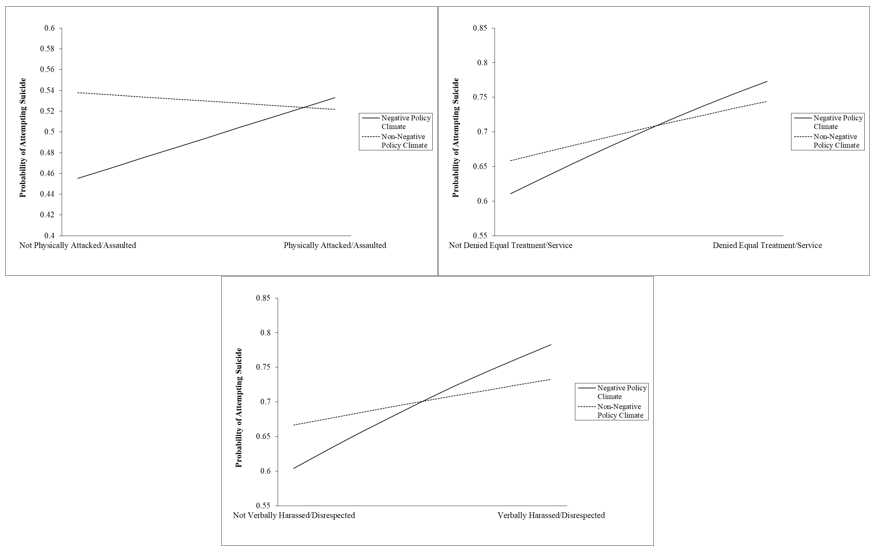

Supplement: Supplementary file 2 — (61.0 KB DOCX) [file 11121_2025_1825_MOESM2_ESM.docx]
